# Analysis of multiple chromosomal rearrangements in the genome of *Willisornis vidua* using BAC-FISH and chromosome painting on a supposed conserved karyotype

**DOI:** 10.1186/s12862-021-01768-y

**Published:** 2021-03-02

**Authors:** Talita Fernanda Augusto Ribas, Julio Cesar Pieczarka, Darren K. Griffin, Lucas G. Kiazim, Cleusa Yoshiko Nagamachi, Patricia Caroline Mary O´Brien, Malcolm Andrew Ferguson-Smith, Fengtang Yang, Alexandre Aleixo, Rebecca E. O’Connor

**Affiliations:** 1 Laboratório de Citogenética, Centro de Estudos Avançados da Biodiversidade, Instituto de Ciências Biológicas, Universidade Federal do Pará, Belém, Brazil; 2 School of Biosciences, University of Kent, Canterbury, UK; 3 Cambridge Resource Centre for Comparative Genomics, Department of Veterinary Medicine, University of Cambridge, Cambridge, UK; 4 Cytogenetics Facility, Wellcome Trust Sanger Institute, Hinxton, UK; 5 Finnish Museum of Natural History, University of Helsinki, Helsinki, Finland

**Keywords:** Antbirds, BAC clones, Chromosome painting, Cytogenetics, Rearrangements

## Abstract

**Background:**

Thamnophilidae birds are the result of a monophyletic radiation of insectivorous Passeriformes. They are a diverse group of 225 species and 45 genera and occur in lowlands and lower montane forests of Neotropics. Despite the large degree of diversity seen in this family, just four species of Thamnophilidae have been karyotyped with a diploid number ranging from 76 to 82 chromosomes. The karyotypic relationships within and between Thamnophilidae and another Passeriformes therefore remain poorly understood. Recent studies have identified the occurrence of intrachromosomal rearrangements in Passeriformes using *in silico* data and molecular cytogenetic tools. These results demonstrate that intrachromosomal rearrangements are more common in birds than previously thought and are likely to contribute to speciation events. With this in mind, we investigate the apparently conserved karyotype of *Willisornis vidua*, the Xingu Scale-backed Antbird, using a combination of molecular cytogenetic techniques including chromosome painting with probes derived from *Gallus gallus* (chicken) and *Burhinus oedicnemus* (stone curlew), combined with Bacterial Artificial Chromosome (BAC) probes derived from the same species. The goal was to investigate the occurrence of rearrangements in an apparently conserved karyotype in order to understand the evolutionary history and taxonomy of this species. In total, 78 BAC probes from the *Gallus gallus* and *Taeniopygia guttata* (the Zebra Finch) BAC libraries were tested, of which 40 were derived from *Gallus gallus* macrochromosomes 1–8, and 38 from microchromosomes 9–28.

**Results:**

The karyotype is similar to typical Passeriformes karyotypes, with a diploid number of 2n = 80. Our chromosome painting results show that most of the *Gallus gallus* chromosomes are conserved, except GGA-1, 2 and 4, with some rearrangements identified among macro- and microchromosomes. BAC mapping revealed many intrachromosomal rearrangements, mainly inversions, when comparing *Willisornis vidua* karyotype with *Gallus gallus*, and corroborates the fissions revealed by chromosome painting.

**Conclusions:**

*Willisornis vidua* presents multiple chromosomal rearrangements despite having a supposed conservative karyotype, demonstrating that our approach using a combination of FISH tools provides a higher resolution than previously obtained by chromosome painting alone. We also show that populations of *Willisornis vidua* appear conserved from a cytogenetic perspective, despite significant phylogeographic structure.

## Background

The Order Passeriformes is one of the most diverse taxa of birds in terms of phenotypic difference and species richness, with around 6000 species. The group also demonstrates a wide variety of morphological adaptations compatible with their eating habits and ecological niches [1]. In Brazil, there are approximately 1900 species of Passeriformes, with 1300 occurring in the Amazon, of which 265 are endemic [2, 3]. This order represents one of the largest adaptive radiations among vertebrates, with representative species in all continents except Antarctica, and with the most diversity found in the tropics [1].

The Thamnophilidae family (typical antbirds), are a widespread radiation of insectivorous Passeriformes birds. They are a monophyletic and diverse group [4] of species and 45 genera with many occurring in upland forests of the Neotropics [5, 6]. In Brazil there are 195 species of Thamnophilidae birds [7], although it is likely that this is an underestimation of true diversity, particularly in the Amazon region, since the Brazilian avifauna is still being sampled [8]. The Thamnophilidae is a polytypic taxon, meaning that they are likely to be many more species of which we are unaware [8–15]. In Brazil as a whole, there have been 31 new species described in the last decade, 15 of which were found in the Amazon region [17].

A useful tool for characterizing bird species, as well as for understanding their evolutionary history and genome organization is through karyotype analysis [18]. Karyotypic studies can be employed for the detection of rearrangements involved in speciation events in evolutionary comparative studies and could be helpful in defining cryptic species without obviously genetic divergence, but with chromosomal differences [18–20]. Several evolutionary studies involving karyotypic characters have been conducted (reviewed in Griffin et al. [22] and Kretschmer et al. [18]), but most are focussed on poultry due to the ease of obtaining samples, or on birds that are of significant commercial value, such as the Psittacidae, targets of biopiracy [23].

In the context of chromosome evolution, avian karyotypes are considered stable when compared to other vertebrate groups such as mammals [24]. However, as in any group of organisms, distinct evolutionary variations occur, with some species such as the falcons and parrots presenting greatly rearranged karyotypes [24–26]. Avian species, in general have a stable diploid number, where 2n = ~ 80 chromosomes. Despite some species having morphologically similar macrochromosomes, recent studies using chromosome painting, BAC FISH and genome sequencing analyses [25] have shown that some species have a complex pattern of pericentric and paracentric inversions, and many intrachromosomal rearrangements, including micro inversions, fusions and fissions [27–29].

There are five published sets of whole chromosome paints in birds: *Gallus gallus* (Chicken; GGA, 2n = 78, Galliformes) [31], *Burhinus oedicnemus* (Eurasian Stone Curlew, BOE, 2n = 42, Charadriiformes) [32], two species of Accipitriformes, *Leucopternis albicollis* (White Hawk, LAL, 2n = 66) [33], and *Gyps fulvus* (Griffon Vulture, GFU, 2n = 66) [34], and *Zenaida auriculata* (Eared Dove, ZAU, 2n = 76) [35]. Of these, *Gallus gallu*s is the only species that has its whole genome sequenced [36]. *Gallus gallus* derived paints have been hybridized to more than 40 bird species from diverse families, providing maps of reliable chromosome homologies (reviewed in [22, 37, 38]. These results suggest that the ancestral karyotype of birds is similar to the *Gallus gallus* karyotype. Only 21 species of Passeriformes, most belonging to the Sub-Order Oscines, have been investigated by chromosome painting to date [28, 38–46], and only the Wedge-Billed Woodcreeper (*Glyphorynchus spirurus*) was investigated using *Burhinus oedicnemus* probes [7]. The phylogenetic relationship among *Gallus gallus*, zebra finch, *Burhinus oedicnemus* and the Wedge-Billed Woodcreeper are summarized in Additional file 1 based in Prum et al. [48].

Cytogenetic analysis of birds in Brazil began in the 1960 s with descriptive studies that summarized the karyotypes of about 200 species of birds, representing about 14 % of the country’s bird life at that time [49, 50]. Our cytogenetic understanding of Brazilian avifauna has gradually increased, however finding even the most basic information, such as diploid number and chromosome morphology, can be challenging. Despite there being approximately 200 species in the Thamnophilidae, there is little cytogenetic information available for this family. Just four species of Thamnophilidae have been karyotyped: *Pyriglena leucoptera*, 2n = 82 [51], *Isleria hauxwelli*, 2n = 80 [52], *Thamnophilus doliatus*, 2n = 82 [53], and *Dysithamnus mentalis*, 2n = 76 [52]. *Willisornis vidua*, the Xingu Scale-backed Antbird, is an interesting species to be evaluated for their karyotypic evolution as the species is commonly found and widely distributed across the Amazonian region as well as being a polytypic taxon whose subspecies distribution seem to be restricted to boundaries of major rivers in the Amazon basin and plumage differences [14].

Taking these factors into account and given that the identification of chromosomal inversions cannot be visualised using chromosome painting alone, in this study we used a combination of BACs (Bacterial Artificial Chromosomes) and comparative genomic mapping with chromosome paints of *Gallus gallus* and *B. oedicnemus* to investigate the occurrence of rearrangements in an apparently conserved karyotype of *Willisornis vidua*. Our main goal is to use these techniques to better understand the evolutionary history and taxonomy of this species.

## Results

### Karyotypic description and chromosome painting

**Fig. 1:**
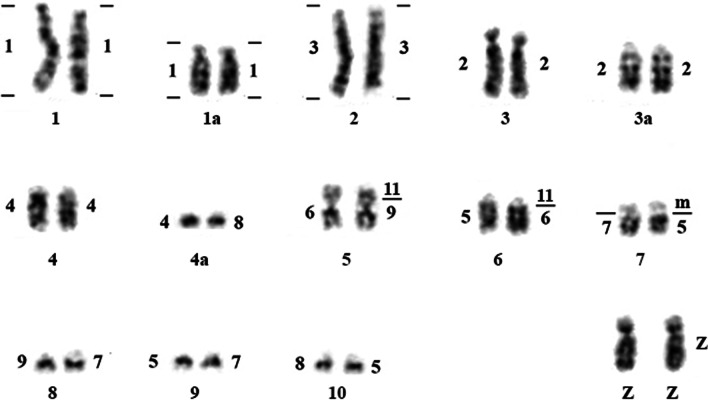
G-banding karyotype of* Willisornis vidua* showing the localization of the corresponding probes. *Gallus gallus* is showed in the left and *Burhinus oedicnemus* in the right. We follow the nomenclature of Nie et al. [35] for GGA probes

Both *Willisornis vidua* subspecies have karyotypes indistinguishable from each other with a diploid number of 80, being comprised mostly of acrocentric chromosomes (chromosomes 7 and 9 are metacentric). The Z chromosomes are submetacentric. We have not identified the W chromosome morphology since only male specimens were analysed (Fig. 1).

**Fig. 2:**
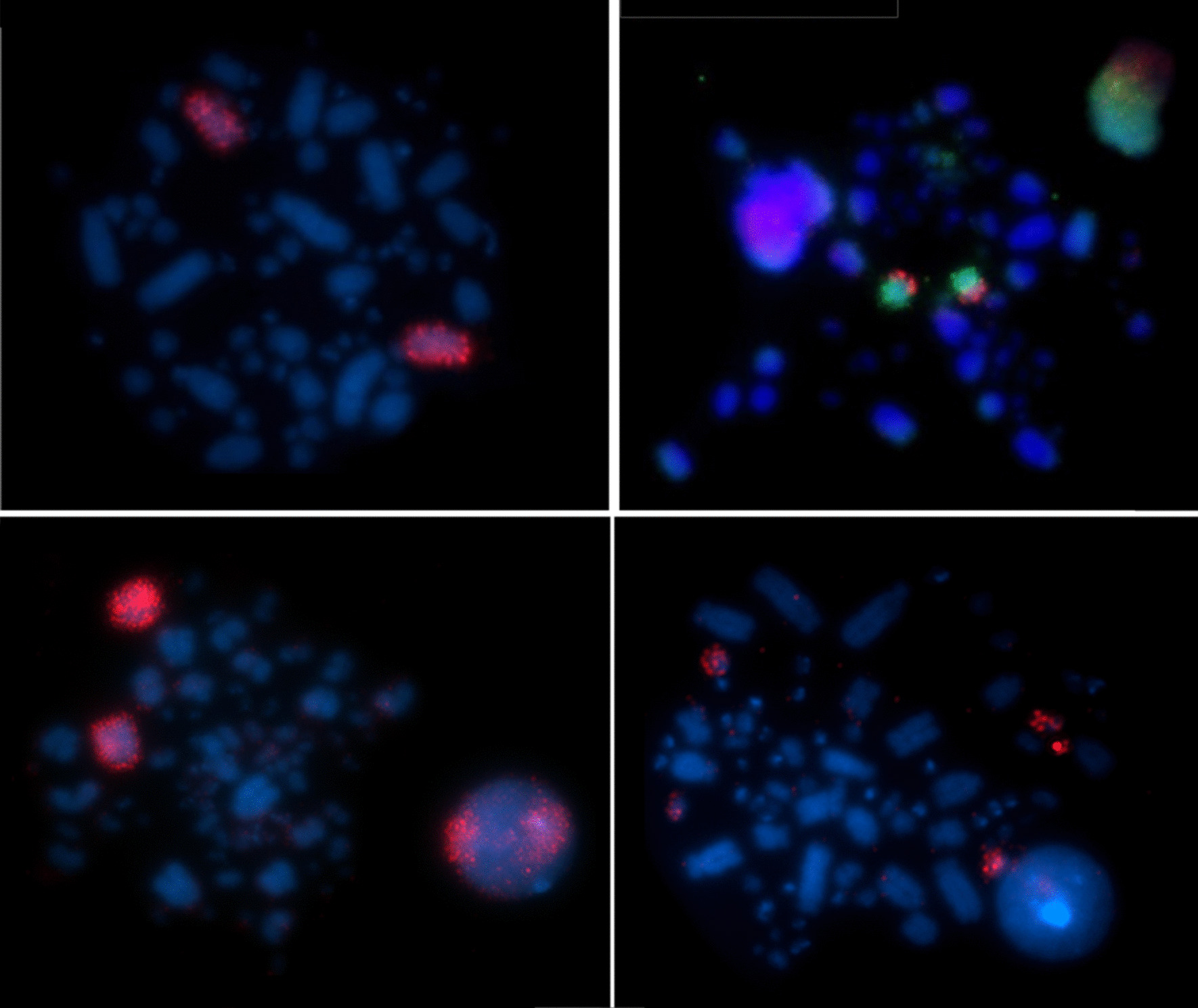
Examples of chromosome painting in *Willisornis vidua* with *Burhinus oedicnemus* chromosomes probes. BOE-Z (left, above), BOE11 Texas red / 9 FITC (right, above), BOE3 (left, below) and BOE-14 (right, below)

Hybridisation of BOE whole chromosome probes reveal 18 homologous segments on macrochromosomes, including Z, and give 19 signals on microchromosomes on the *Willisornis vidua genome* (Fig. 2; Table 1). Only five probes (BOE-3, 4, 6, 9 and Z) show preserved synteny in eight chromosomes of WVI. The other probes show two or more hybridisation signals in WVI chromosomes. Hybridisation of GGA whole chromosome probes reveal 14 homologous segments on *Willisornis vidua* macrochromosomes. The correspondence between BOE, GGA and WVI karyotypes are shown in Table 1. We used data from Nie et al. [32] to identify homologies with GGA.

**Table 1:** Chromosomal correspondence among *Burhinus oedicnemus*, *Gallus gallus* according to [32] and *Willisornis vidua*, revealed by FISH with *Burhinus oedicnemus* chromosome-specific paints

*Burhinus oedicnemus* Chromosome	*Gallus gallus* homologue	*Willisornis vidua* homologue
1	1	1, 1a
2	2	3, 3a
3	3	2
4	4q	4
5	7, 8	7, 9
6	5	6
7	9, 2 micros	8, 9
8	4p, 1 micro	2 micros
9	2 micros	7p, 1 micro
10	2 micros	2 micros
11	2 micros	5pd, 1 micro
12	2 micros	2 micros
13	2 micros	6pd, 7pd, 1 micro
14	2 micros	2 micros
15 + 16	3 micros	3 micros
17 + 18 + 19 + 20	1 micro	4 micros
Z	Z	Z, 1 micro

*micro *microchromosome, *p *short arm, *d *distal

### BAC-FISH

**Fig. 3:**
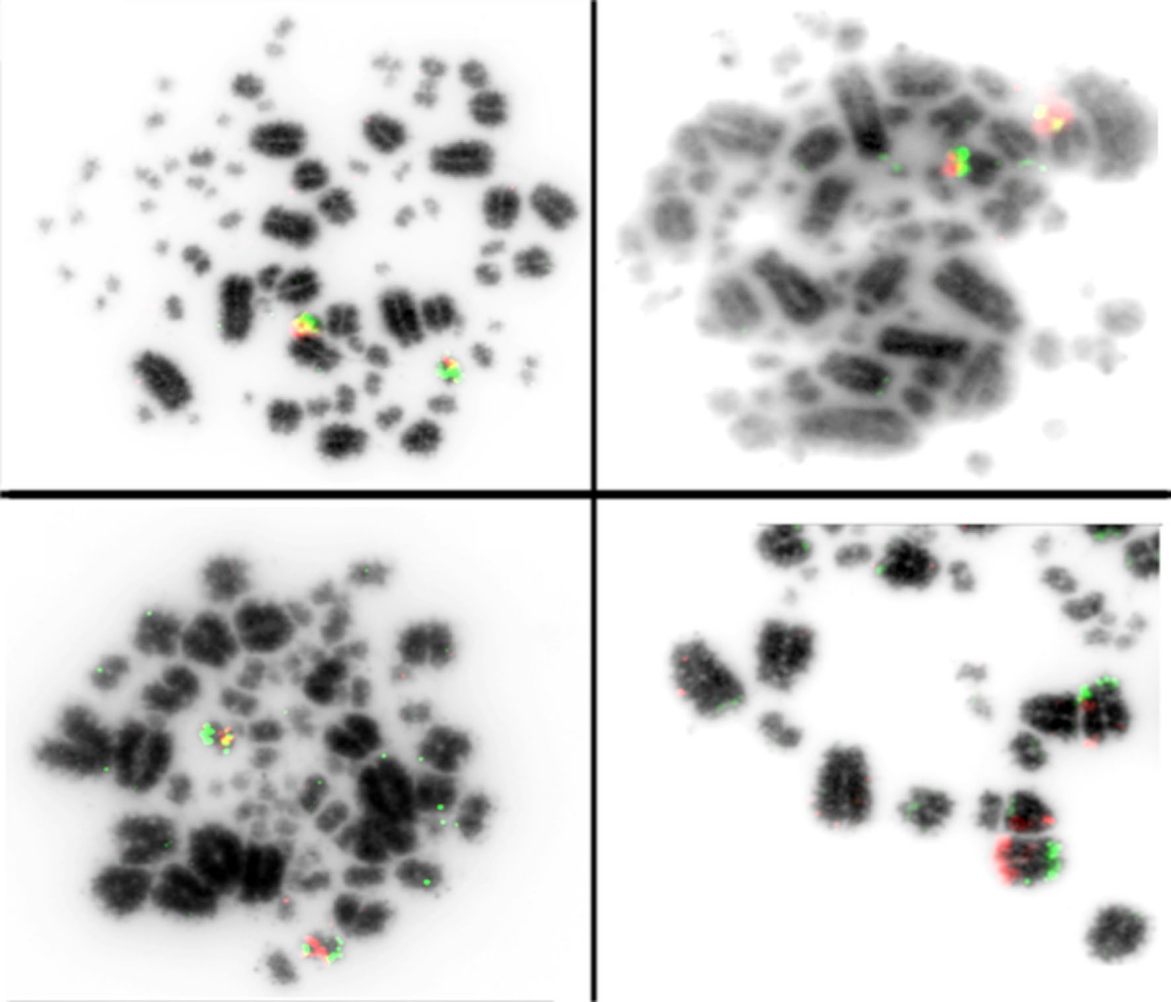
Examples of BAC-FISH microchromosomes and Z in the *Willisornis vidua* karyotype. The micros correspond to CH261-121N21 F with 154H1 Tx (GGA11; left, above), (TGMCBA-375I5 F with CH261-42P16 (TGA17/GGA17; right, above), CH261-115I12 F with TGMCBA-321B13 Tx (GGA13/TGA17; left, below) and TAG-Z TGMCBA-200J22 F with 270I9 Tx (TGAZ; right, below)

Comparative mapping of BAC clones in *Willisornis vidua*, using *Gallus gallus* and *Taenopygia guttata* BACs, reveal a total of 43 hybridisation signals in the *Willisornis vidua* genome. We also hybridised the microchromosome BAC clones corresponding to microchromosomes 9–28 in the *Gallus gallus* (Fig. 3).

**Fig. 4:**
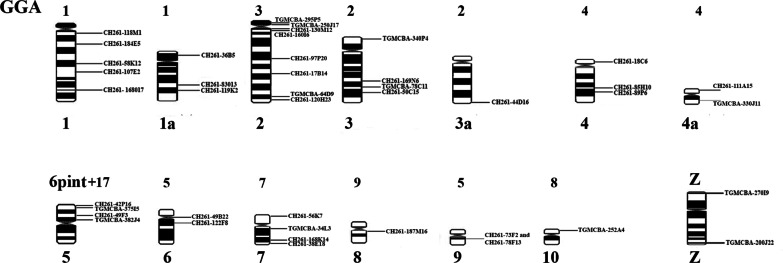
Standard partial ideogram of *Willisornis vidua* male based on G-banding. The BAC mapping is on the right side, revealing 42 rearrangements between GGA, TGU and WVI

**Fig. 5:**
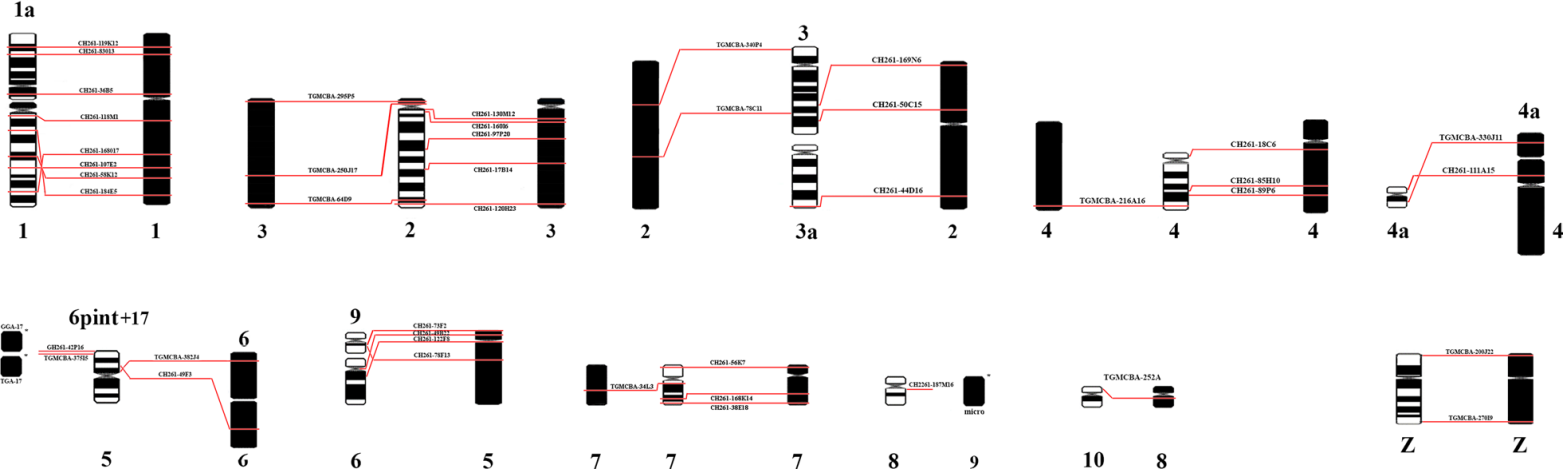
Comparative genomics of *Willisornis vidua*, *Gallus gallus* and *Taeniopygia guttata*. The chromosomes compared are 1–9 and micro 17, revealing 42 interchromosomal and 9 intrachromosomal rearrangements

**Fig. 6:**
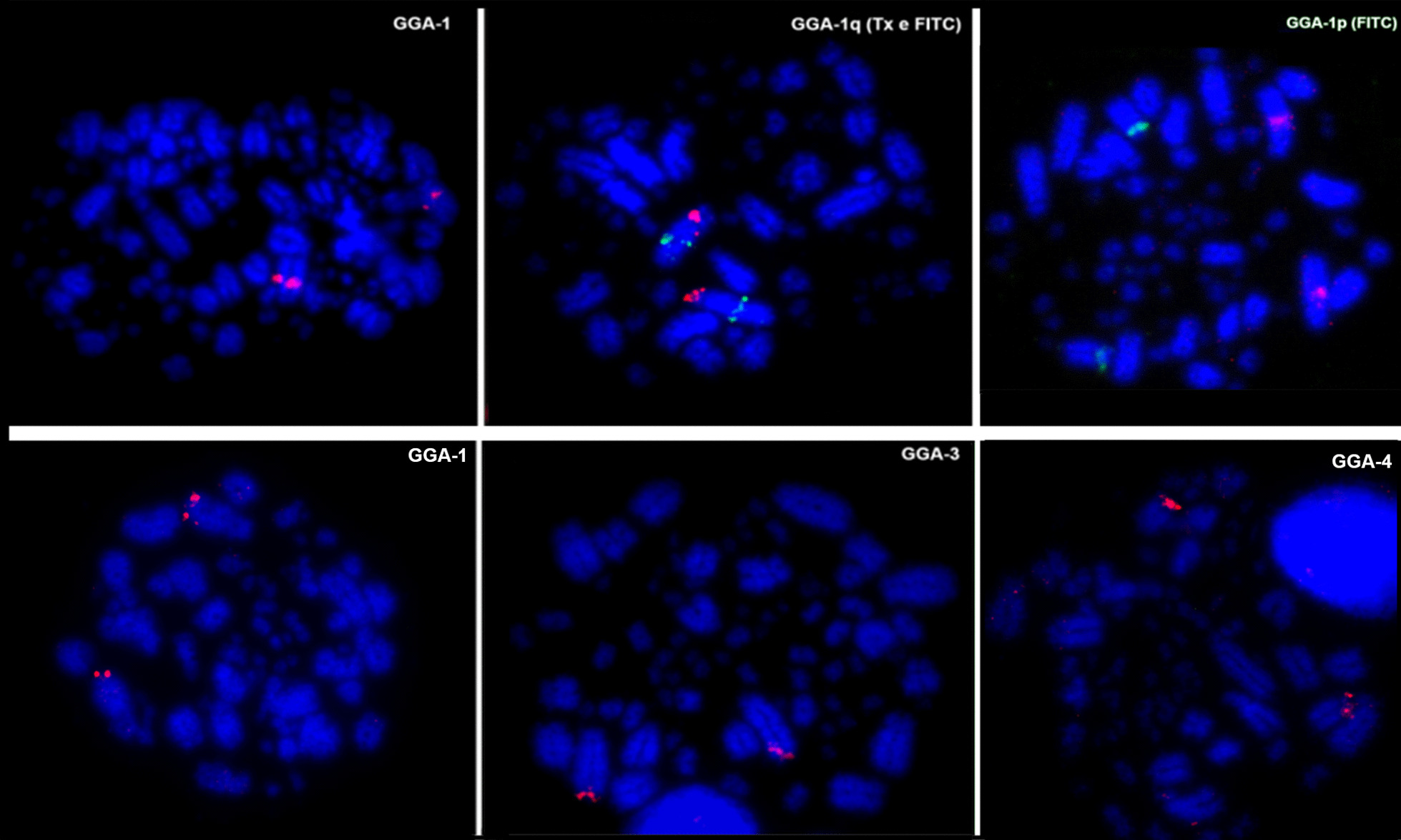
Examples of BAC-FISH macrochromosomes in *Willisornis vidua* karyotype. The BACs mapped are CH261-1845 (GGA1; left, above), CH261-36B5 F with 58K12 Tx (GGA1; middle, above), CH261-107E2 F with 118M1 Tx (GGA1; right, above), CH261-83O13 (GGA1; left, below), CH261-1203H23 (GGA3; middle, below) and (GCH261-18C6 GA4; right, below). F = Fitc Tx = Texas red

Using these 43 BACs, we find 29 intrachromosomal differences between the three species. We also report 14 interchromosomal differences in *Willisornis vidua* when compared to *Taeniopygia guttata* and *Gallus gallus*, using the latter as the reference (Figs. 4 and 5; Table 2). Examples of hybridisation with BAC clones are shown in Figs. 6. We observed extensive homology between GGA and WVI chromosomes. The differences are in *Gallus gallus* microchromosome 9, which hybridised to WVI macrochromosome 8, and the homolog of micro 17 in GGA and TGA, which hybridised to the distal region of WVI macro 5 (Fig. 3; Table 2).

**Table 2:** List of BACs for GGA chromosomes and the corresponding chromosomes in *Willisornis vidua*

BAC Clone	WVI	GGA/TGU	Orientation	Rearrangements
CH261-119K2	1a	1	1	Fusion
CH261-83O13	1a	1	2	Fusion
CH261-36B5	1a	1	3	Fusion
CH261-118M1	1	1	4	Inversion
CH261-168O17	1	1	8	Inversion
CH261-107E2	1	1	7	Inversion
CH261-58K12	1	1	6	Inversion
CH261-184E5	1	1	5	Inversion
TGMCBA-340P4	3	2	1	Inversion
CH261-169N6	3	2	2	Inversion
CH261-50C15	3	2	4	Inversion
TGMCBA-78C11	3	2	3	Inversion
CH261-44D16	3a	2	5	Inversion
TGMCBA-295P5	2	3	1	Fusion
TGMCBA-130M12	2	3	3	Inversion
CH261-160I6	2	3	4	Inversion
CH261-97P20	2	3	5	Inversion
CH261-17B14	2	3	6	Inversion
CH26-250J17	2	3	2	Inversion or translocation
TGMCBA-64D9	2	3	7	Inversion
CH261-120H23	2	3	8	Fusion
TGMCBA-330J11	4a	4	2	Inversion
CH261-111A15	4a	4	1	Inversion
CH261-18C6	4	4	3	Inversion
CH261-85H10	4	4	4	Inversion
CH261-89P6	4	4	5	Fusion
TGMCBA-216A16	4	4	6	Fusion
CH261-73F2	6	5	1	Inversion
CH261-49B22	6	5	3	Inversion
CH261-122F8	9*	5	4	Inversion
CH261-78F13	9*	5	2	Inversion
TGMCBA-382J4	5	6	2	Inversion
CH261-49F3	5	6	1	Inversion
CH261-56K7	7	7	1	Fusion
TGMCBA-34L13	7	7	2	Inversion
CH261-186K14	7	7	3	Inversion
CH261-38E18	7	7	4	Fusion
TGMCBA-252A4	10	8	1	Inversion
CH261-187M16	8	9	1	Fusion
CH261-42P16	6p distal	17	2	Fusion
TGMCBA-375I5	6p distal	17	1	Fusion
TGMCBA-200J22	Z	Z	1	Fusion
TGMCBA-270I9	Z	Z	2	Fusion

*Probes overlapped in the fish images **The correct position of the micro involved is beyond the scope of this paper due to the absence of the WVI genome; The BACs used are those previously developed by Damas et al. [25]. The orientation means the order of the BAC clone in WVI karyotype

## Discussion

### Karyotypic variation and chromosome painting

Here we describe, for the first time, cytogenetic analysis of the species *Willisornis vidua*, collected from the Brazilian Amazon. All subspecies represented here have karyotypes that are similar to the typical avian karyotype with few macrochromosomes, and many microchromosomes.

Chromosome painting with BOE and GGA probes show that most GGA homologues are conserved *in toto* with the exception of GGA1, 2 and 4. GGA4 is homologous to WVI 4 plus a microchromosome (Fig. 1), as seen in most avian species where the *Gallus gallus* is a rare exception (see Griffin et al. [22]). On the other hand, the fission of the GGA1 homologue is considered a signature pattern seen in Passeriformes [29, 45, 53–55], and the current work found the same arrangement. This fission is not however a synapomorphy confined only to Passeriformes birds, as it is present also in the phylogenetic branch comprising Strigiformes, Passeriformes, Columbiformes and Falconiformes [22, 34, 41, 46, 57]. On the other hand, the fission of GGA1 found by FISH also corroborates the recent hypotheses that Passeriformes and Psittaciformes are sister-groups [18, 48, 58]. Further research, including multicolor banding probe sets and BAC-based multicolor barcoding may test if this hypothesis could be homoplasy or a real signature of this group.

Amazonian rivers are considered geographical barriers for some Amazonian taxa, including birds, thus contributing to speciation events [13, 58–61]. However, the populations of *Willisornis vidua vidua* and *Willisornis vidua nigrigula* sampled herein are separated by the Xingu and Tocantins rivers, and no chromosomal differences were detected, despite a previous study having detected significant genetic variation showing that they are not sister groups [63]. Therefore, here we show that populations of *Willisornis vidua* appear conserved from a cytogenetic perspective, despite significant phylogeographic structure. Future studies should address whether *Willisornis vidua* and the remaining species in the genus *Willisornis* (*W. poecilinotus*) exhibit any heterotic negative rearrangements in populations with high levels of genetic variation [64, 65]. Both species are in direct contact in the headwaters of the Tapajós and Xingu rivers, where they intergrade, although at a low frequency, and with highly introgressed hybrids showing low fitness coefficients [66].

### BAC-FISH

The results presented here show conservative evolutionary stability in microchromosomes when compared with *Gallus gallus* and *Taeniopygia guttata*, which is consistent with the genomic and karyotypic conservation in the avian Class [25, 66–68]. Exceptions were found with *Gallus gallus* microchromosome 9, which hybridised to WVI chromosome 8, and the homolog of chromosome 17 in GGA and TGU, which hybridised to the distal region of WVI chromosome 5 (Fig. 3). Our results therefore demonstrate the advantages of using both chromosome painting and BAC-FISH techniques together. By using both, we did not find any evidence of reciprocal translocation, although the data presented here demonstrate that this approach can identify multiple intrachromosomal rearrangements between species (Figs. 4 and 5).

O’Connor et al. [69] compared the *Gallus gallus* with the *Apalone spinifera* (Spiny softshell turtle), using chromosome painting and BAC-FISH, and discovered that they too have similar karyotypic patterns, with most chromosomes being precise counterparts of each other. Here, we also used both methods to show the precise definition of homologies between *Willisornis vidua*, *Burhinus oedicnemus* and *Gallus gallus*. This definition, first detected by chromosome painting, was confirmed by BAC-FISH which also revealed the intrachromosomal rearrangements in *Willisornis vidua*.

We recently proposed the hypothesis that the GGA2 fission is a synapomorphic trait unique to the Furnariidae family as it is present in both *Glyphrorynchys spirirus* [46] and *Synallaxis frontalis* [70]. The fission of GGA2 also appears to be present in the karyotype of two species of Formicariidae (sister-group of Furnariidae) [51, 71]. As this rearrangement is ancestral [22], the GGA2 fission was considered to be a homoplasy in Suboscines. However, since several species of this suborder have been analysed by chromosome painting [46, 47, 70] presented here and three species of Thamnophilidae (Ribas et al., data in preparation), together with one species of the Tyrannidae family [47], we suggest that this rearrangement could be a synapomorphic trait in Suboscines. Studies using chromosome-level assemblies are required to test the exact site of the chromosomal breakpoints and confirm this suggestion.

O’Connor et al. [72] found extensive chromosomal rearrangements in the *Falco cherrug* (saker falcon) when compared with the *Gallus gallus*. They found 36 intrachromosomal differences and 12 fissions and 5 fusions between the two species. Here, we found 29 intrachromosomal rearrangements, demonstrating that despite having an apparently conserved karyotype the *Willisornis vidua* genome is highly rearranged intrachromosomally.

The most conserved chromosome found here was the Z. This chromosome showed the same pattern as the *Gallus gallus* Z when compared with *Taeniopygia guttata* probes (Figs. 3 and 5). The Z conservation was expected since this chromosome showed uniform painting pattern with *Gallus gallus* painting probes [18, 37, 46] and seems conserved for the last < 80 million years of bird evolution, reflecting the remarkable stability of the avian karyotype. The Z chromosome demonstrates variable morphology among the Thamnophilidae birds when compared with other species in this family (data in preparation), being acrocentric in *Phlegopsis*, *Isleria* and *Myrmotherula*, submetacentric in *Thamnophilus*, *Pyriglena* and *Willisornis vidua*, and metacentric in *Thamnomanes*. Chromosomal variations in the Z chromosome are attributed to pericentric inversions and/or centromeric repositioning. In an evolutionary context, some rearrangements in the Z found here and shared with these species suggest a common ancestral.

Besides the high diploid number, we did not find any evidence of microchromosome fission in *Willisornis vidua*. Comparison of the *Willisornis vidua* karyotype with the PAK suggests that the 2n = 80 is the result of macrochromosome fission, and that the ancestral microchromosome pattern is consistent with that found in Passeriformes and many other orders [69]. Other species of the Thamnophilidae family need to be analysed to test the hypothesis raised here.

## Conclusions

We describe, for the first time, the *Willisornis vidua* karyotype and its chromosomal homology map with *Gallus gallus* and stone curlew using chromosome painting probes which reveals an apparently conserved karyotype. We also suggest that based on the data presented here and in previous studies, the GGA2 split is a synapomorphic trait in the Suboscines suborder, given that it was found in the Tyrannidae, Furnariidae and Thamnophilidae families.

We also found a series of new intrachromosomal rearrangements in these species when compared to the *Gallus gallus* and *Taeniopygia guttata* genomes (using BAC probes) that are beyond the resolution of chromosome painting. Further research comparing additional species and additional orders based on chromosome-level genome mapping will provide greater understanding about the mechanisms and patterns of chromosome rearrangement among these related species, and will provide greater clarity over whether the rearrangements found here could be synapomorphic traits of the Thamnophilidae family or autapomorphies of *Willisornis vidua*.

## Methods

### Samples and chromosomal preparation

Three specimens of *Willisornis vidua* were collected with mist nets from natural populations of the Brazilian Amazon in Belém and Tapajós endemism areas in the municipalities of Belterra (2°24’05’’S/55°04’40’’W), Mocajuba (2°39’53.7’’S/49°35’’18.3’’W) and Santa Bárbara (1°12’14"S/48°17’39"W) in Pará state. The three specimens correspond to two subspecies: *Willisornis vidua* from Belterra-Pa is *Willisornis vidua nigrigula* and the two others specimens of *Willisornis vidua from* Mocajuba-Pa and Santa Barbará-Pa are *Willisornis vidua vidua*, whose geographic distributions are limited by the Xingu River, a major tributary of the Amazon Rivers and which acts as a geographical barrier between these subspecies. These subspecies were distinguished by their geographic distribution and plumage color [14]. The species sampled are not endangered or protected.

After capture in the field with mist nets, specimens were maintained in the lab with food and water, free from stress, until their necessary euthanasia. At the lab, colchicine treatment was performed according to the weight of the bird and after 30 minutes to 1 hour. The euthanasia was made with intraperitoneal injection of buffered and diluted barbiturates (86 mg/kg) after anaesthesia with ketamine (40 mg/kg), following The American Veterinary Medical Association Guidelines for the Euthanasia of Animals. The bone marrow preparations obtained from the femur were performed according to Garnero and Gunski [73], with modifications.

Voucher specimens were deposited in the bird collection of the Museu Paraense Emilio Goeldi. JCP has a permanent field permit number 13,248 from “Instituto Chico Mendes de Conservação da Biodiversidade”. The Cytogenetics Laboratory from UFPa has a special permit number 19/2003 from the Brazilian Ministry of Environment for samples transport and 52/2003 for using the samples for research.

### Determination of the karyotype

To determine diploid numbers and generate karyotypes, fifty metaphase spreads from each bird subspecies were stained with a combination of inverted DAPI pattern (black/white) or G banding. The chromosomes are displayed according to the *Taeniopygia guttata* karyotype [46]. Measurement analysis allowed the construction of an idiogram. Bands were divided into “light” (pale on G-banding), “dark” (dark on G-banding) and “grey” (bands not distinguishable).

### Whole chromosome probes prepared for chromosome painting

For chromosomal painting studies we used kits produced at the Cambridge Resource Centre for Comparative Genomics, Department of Veterinary Medicine, University of Cambridge, UK, by separation of whole chromosomes using flow cytometry (chromosomes 1–9 of *Gallus gallus* - GGA - and all chromosomes of *Burhinus oedicnemus* - BOE). From the products of the primary PCR performed to amplify the DNA of the isolated chromosomes, a second round of DOP-PCR, using 1 µl of product, allowed its labelling with Cy3-dUTP, biotin-16-dUTP (Boehringer Mannheim) or fluorescein isothiocyanate − 12-dUTP (Amersham), subsequently detected with avidin-FITC or avidin-FITC.

For the hybridization experiments, metaphase chromosome preparations were aged for 1 h at 65 °C and treated in 1 % pepsin for 5 min. Chromosomal DNA was denatured at 60 °C in 70 % formamide for 30 seconds. The probes were denatured under the same conditions. The probes were hybridized for three days at 37 °C. After that the slides were washed twice in formamide 50 %, 2xSSC, and once in 4xSSC/Tween at 40˚C. For visualization of the biotin-labelled probes a layer of Cy3-a or Cy5-avidin (1:1000 dilution; Amersham) was used. For FITC-labelled probes we used a layer of rabbit anti-FITC (1:200; DAKO). Slides were mounted in a mounting medium with DAPI called Vectashield (Vector Laboratories) [32].

### Generation of Labelled FISH probes for BAC-FISH

#### Selection of BAC clones

The clones selected for mapping experiments were originally obtained from the BACPAC Resource Centre at the Children’s Hospital Oakland Research Institute and the zebra finch TGMCBa library (Clemson University Genomics Institute). The full set of BAC clones reported in Damas et al. [25] as suitable for inter-species hybridization in birds were used for hybridization. In total, 78 probes from the *Gallus gallus* and *Taeniopygia guttata* BAC libraries were tested, of which 40 correspond to GGA and TGA macrochromosomes 1–8 and 38 to GGA and TGA microchromosomes 9–28.

#### Preparation of BAC clones for FISH

Briefly, BACs were cultured in Luria Bertani Agar (LB Agar) and the clone DNA was extracted by QIAprep Spin Miniprep Kit (Qiagen). BACs were labelled by nick translation using Texas red-12-dUTP (Invitrogen) and FITC-fluorescein-12-UTP (Roche) prior to purification using the Qiagen nucleotide removal kit [25].

#### Slide preparation for BAC FISH

Chromosome suspensions were dropped on each half of the slide and allowed to air dry. Slides were washed in 2xSSC (Saline-Sodium Citrate) (Gibco) for 2 minutes, dehydrated by serial ethanol washing for 2 minutes each in 70 % (v/v), 85 % and 100 % ethanol and left to air dry.

### Fluorescence in situ hybridization for BAC FISH

The probe mix was prepared by adding 1.5 µl of FITC labelled probe, 1.5 µl of Texas Red labelled probe, 1 µl of *Gallus gallus* Hybloc (Applied Genetics Laboratories), 6 µl of Hyb I (Cytocell) hybridisation buffer to a total volume of 10 µl and a probe concentration of 10 ng/µl. Slides were incubated in a hybridisation chamber at 37 °C for 72 hours and then the slides were washed with 2xSSC with 0.05 % of Tween-20 (Sigma-Aldrich) and stained with Vectashield Antifade Mounting Medium with DAPI (Vectorlab).

### Microscopy

Metaphase images were captured using an Olympus BX-61 epifluorescence microscope equipped with a cooled CCD camera and SmartCapture 3 software (Digital Scientific UK). Three different filters were used to acquire images with DAPI, fluorescein isothiocyanate and Texas Red fluorochromes.

## Supplementary information

**Addtional file 1**. Phylogenetic relationship among chicken, zebra finch, the Eurasian Stone Curlew and the Wedge-Billed Woodcreeper. The phylogeny is based in Prum et al. [48].

## Data Availability

All relevant data used in this study are found in the paper.

## Abbreviations

BAC: Chromosomal Artificial of Bacteria
BOE: B*urhinus oedicnemus*
DOP-PCR: Degenerated oligonucleotide-primed polymerase chain reaction
dUTP: Deoxyuridine triphosphate
FISH: Fluorescent in Situ Hybridisation
GGA: *Gallus gallus*
TGU: *Taeniopygia guttata*
WVI: *Willisornis vidua*
