# Supplementary figures and images for: Analysis of multiple chromosomal rearrangements in the genome of Willisornis vidua using BAC-FISH and chromosome painting on a supposed conserved karyotype
Source: BMC Ecol Evol. 2021 Mar 2;21:34. doi: 10.1186/s12862-021-01768-y (PMC7927240; doi:10.1186/s12862-021-01768-y)

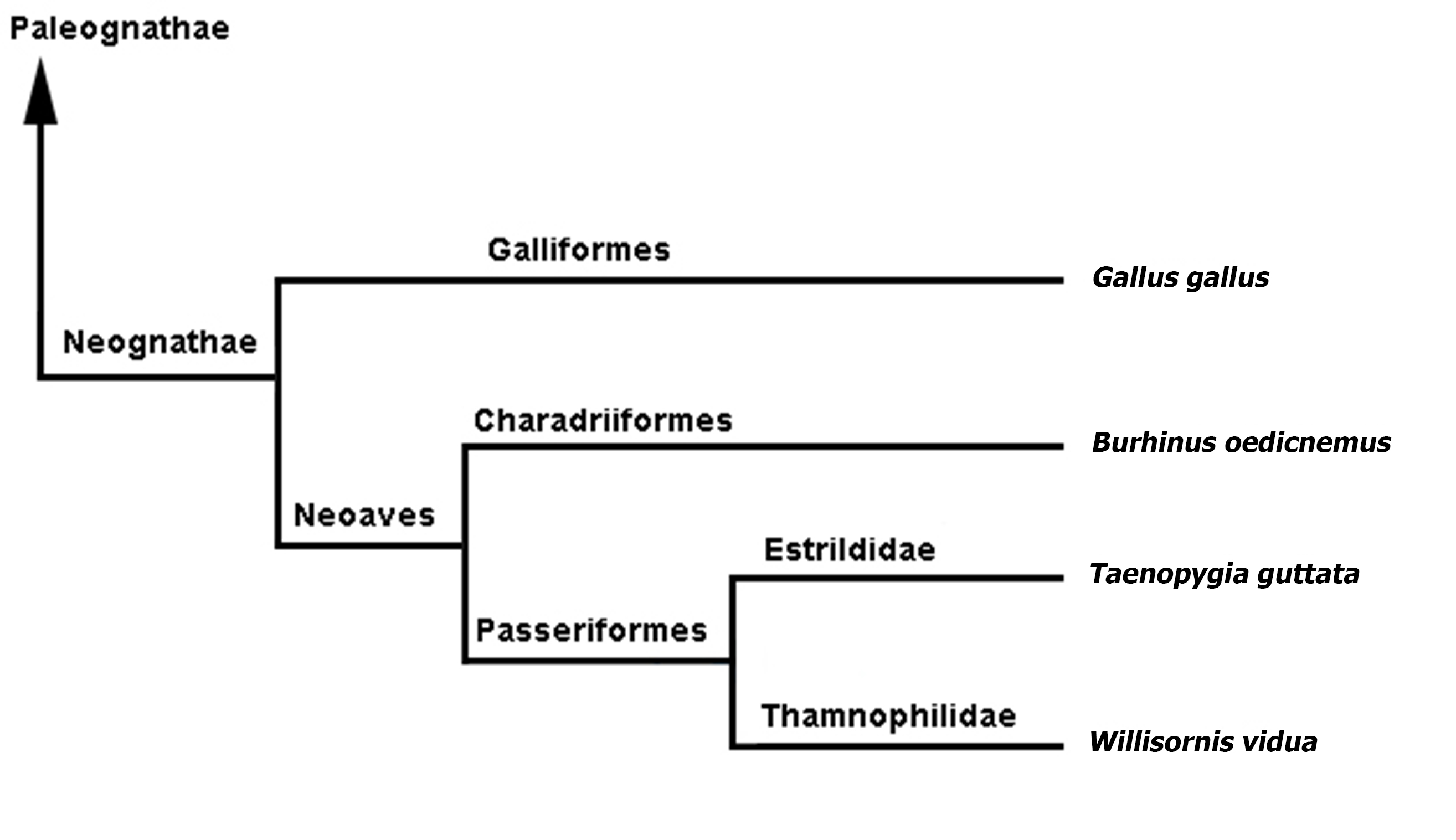

Supplement: Supplementary file 1 — Addtional file 1. Phylogenetic relationship among chicken, zebra finch, the Eurasian Stone Curlew and the Wedge-Billed Woodcreeper. The phylogeny is based in Prum et al. [48]. [file 12862_2021_1768_MOESM1_ESM.tif]
